# Decreased compliance in the deep and superficial conduit veins of the upper arm during prolonged cycling exercise

**DOI:** 10.14814/phy2.13253

**Published:** 2017-04-24

**Authors:** Anna Oue, Kohei Sato, Marina Yoneya, Tomoko Sadamoto

**Affiliations:** ^1^ Faculty of Food and Nutritional SciencesToyo UniversityGunmaJapan; ^2^ Research Institute of Physical FitnessJapan Women's College of Physical EducationTokyoJapan

**Keywords:** Conduit vein, cross‐sectional area, cuff deflation protocol, ultrasound technique

## Abstract

We examined whether there is a difference in compliance between the deep and superficial conduit veins of the upper arm in response to prolonged exercise. Eight young men performed cycling exercise at 60% of peak oxygen uptake until rectal temperature had been increased by 1.1°C for 38–48 min. The cross‐sectional area (CSA) of the brachial (deep) and basilic (superficial) veins was assessed by ultrasound during a cuff deflation protocol. Compliance (CPL) was calculated as the numerical derivative of the cuff pressure and CSA curve. During prolonged exercise, CPL in both conduit veins was similarly decreased when compared with pre‐exercise values; however, the CSA decreased in the deep vein but increased in the superficial vein. In addition, passive heating caused an analogous change in CSA and CPL of superficial vein when compared with prolonged exercise, but did not change CSA and CPL of deep vein. Cold pressor test induced the decreased CSA of deep and superficial veins without the alteration of CPL of both veins. These results suggest that CPL in the deep and superficial conduit veins adjusts to prolonged exercise via different mechanisms.

## Introduction

Veins are highly compliant and contain a large amount of the blood volume during rest (Greenfield and Patterson 1956; Morris et al. 1974). When physiological stress occurs, such as exercise and exposure to heat, the venous system must adjust to this new state, and usually does so by a change in venous compliance (Rothe 1986), which could facilitate the shift of blood from vein to the heart and might allow us to maintain the central blood volume and blood pressure. During prolonged exercise, blood flow increases in not only exercising muscle area but also in non‐exercising skin area in order to restrain the excessive elevated body temperature (Johnson et al. 1974; Wenger et al. 1975; Johnson and Park 1981; Kellogg et al. 1991). In addition, in non‐exercising upper limb, blood flow in muscle area is opposite to that in skin area during prolonged cycling exercise (Johnson and Rowell 1975). These suggest that prolonged exercise especially needs many amount of blood when compared with resting condition, and if these controls failed, exercise was stopped and finally the risk for heatstroke was higher. Thus, the alteration of venous compliance in muscle and skin areas in non‐exercising upper limb during prolonged exercise is likely to play, in part, an important role of homeostatic regulation. However, the adjustments in venous compliance in response to exercise are not fully understood. Venous occlusion plethysmography (VOP) has been used to calculate venous compliance from the relationship between whole‐limb volume changes and the cuff pressure applied on the extremities (Halliwill et al. 1999; Monahan and Ray 2004; Hernandez and Franke 2005; Young et al. 2006). However, VOP has some limitations. Firstly, VOP cannot measure compliance in individual veins. Secondly, since VOP measures changes in whole‐limb volume in the extremity, the data obtained can be erroneous if a volume shift (i.e., capillary filtration) occurs during measurement (Gamble et al. 1993; de Groot et al. 2005). Recent studies (de Groot et al. 2005; Young et al. 2008; Zachrisson et al. 2011) have demonstrated that direct assessment of a large conduit vein using high‐resolution ultrasound is able to eliminate the effects of fluid shift during both rest and sympathoexcitatory maneuvers. However, the changes in compliance that occur in conduit veins during prolonged exercise have not yet been investigated.

Large conduit veins in a limb are roughly divided into two different types: deep and superficial. A deep conduit vein runs near the muscular layers and carries venous blood, mainly from muscle tissues, while a superficial conduit vein runs near the skin and carries the outflow, mainly from subcutaneous tissues (Roddie et al. 1956; Detry et al. 1972). In addition, there is less sympathetic innervation in the deep veins than in the superficial veins (Abdel‐Sayed et al. 1970; Vanhoutte and Lorenz 1970). In view of these physiological and structural differences between the deep and superficial veins, we hypothesized that there would be a difference in adjustment of compliance during prolonged exercise between the deep and superficial conduit veins. The aim of this study was to determine the cross‐sectional area (CSA) and compliance (CPL) of a single deep conduit vein and a single superficial conduit vein of the upper arm during prolonged cycling exercise using ultrasonography.

## Materials and Methods

### Subjects

Twenty one volunteers participated in this study. The study was approved by the Human Ethics Committee of the Japan Women's College of Physical Education and was conducted in accordance with the tenets of the Declaration of Helsinki. The purpose, procedures, and risks of the study were explained to the subjects, and their informed consent was obtained.

### Experimental design

#### Protocol 1

The purpose of this protocol was to determine CSA and CPL of deep vein and superficial vein of the upper arm during prolonged cycling exercise. Eight male volunteers participated in this protocol. Their mean age, height, weight, and peak oxygen uptake ($\dot{V}O_{2\mathrm{peak}}$) were 21.4 ± 1.1 years, 173.4 ± 1.4 cm, 69.6 ± 2.0 kg, and 55.1 ± 2.5 mL/min/kg, respectively. Subjects refrained from caffeine and alcohol ingestion and hard exercise for 24 h and food intake for 2 h before each exercise session. In addition, before each experiment, subjects had a cup (200 mL) of water to prevent the dehydration. Subjects reported to the laboratory on three occasions, each separated by an interval of 7–10 days. On the first visit, each subject's $\dot{V}O_{2\mathrm{peak}}$ was determined during incremental cycling exercise on sitting position using cycle ergometer (Aerobike 800, Combi, Japan) in an environmental chamber adjusted to an ambient temperature of 24°C–26°C. On the second and third visits, the experiments were performed in a thermoneutral environment (25°C, 40% relative humidity) to evaluate the responses in a deep vein or superficial vein during exercise. Subjects rested for at least 50 min in the pre‐exercise period and then started exercise at an intensity of 60% $\dot{V}O_{2\mathrm{peak}}$ using a semi‐recumbent cycle ergometer (EC‐3700; CatEye Co., Ltd, Osaka, Japan) until rectal temperature increased by 1.1°C from the pre‐exercise value. The duration of exercise was 38–48 min. The rationale for the 1.1 increase was as following. In the case of prolonged exercise, blood flow response of non‐exercising limb depends on not time course but body temperature (Kenney and Johnson 1992; Ooue et al. 2008). In addition, in our pilot study, since the movement of body during exercise was greater as the exercise time was longer (more 50 min), it was difficult to measure the venous compliance accurately. The testing order for second and third visits was randomized.

#### Protocol 2

The purpose of this protocol was to determine the effect of passive heat stress without exercise on CSA and CPL of deep vein and superficial vein of the upper arm. Eight male subjects, who were same people in *protocol 1*, participated in this protocol. Subjects rested in the semi‐recumbent position for 50–60 min under a thermoneutral (25°C, 40% relative humidity; CON) and hot (35°C, 40% relative humidity; HOT) environments. In each CON and HOT, the measurements of deep and superficial veins were randomized between different 4 days. In addition, CON and HOT condition was carried out separately in random order.

#### Protocol 3

The purpose of this protocol was to determine the effect of sympathoexcitation without heat stress on CSA and CPL of deep vein and superficial vein of the upper arm. Thirteen healthy volunteers (three men, 10 women) participated in this study. Their mean age, height, and weight were 21.5 ± 0.5 years, 162.7 ± 4.4 cm, and 53.7 ± 5.5 kg, respectively. After subjects rested in the supine position for at least 20 min in a thermoneutral environment (27.4°C ± 0.2°C), CSA and CPL was measured in the resting condition. And then, subjects submerged a left foot in a bucket of ice and water for 2 min (cold pressor test). The measurements of the deep vein and the superficial vein were randomized between different 2 days.

### Assessment of vein CSA and compliance

The deep vein assessed was the brachial vein and the superficial vein assessed was the basilic vein. For measurement of the CSA, the venous collecting cuff was wrapped around the right upper arm and inflated to 60 mmHg for 8 min, after which the cuff pressure was manually reduced at a rate of 1 mmHg/s from 60 mmHg to 0 mmHg (over 1 min) according to a previously described cuff deflation protocol (Halliwill et al. 1999). In *protocol 1*, the CSA was measured during the final 9 min of the resting period and during the prolonged exercise period. During prolonged exercise, cuff deflation started just when the elevation of rectal temperature achieved 1.1°C. For such occasions, we monitored continuously the rectal temperature, and predicted the timing for the cuff inflation every subjects, because, in pilot study, we confirmed that the rectal temperature during exercise increased linearly with time course of exercise. In *protocol 2*, the CSA was measured during the final 9 min of resting condition in CON and HOT, respectively. In addition, in *protocol 3*, the CSA was measured during the final 9 of resting condition. And in cold pressor test, CSA was measured continuously during cuff deflation protocol, and cold pressor test in each subject was performed from after 7 min of cuff inflation starting to the end of deflation (giving a total cold pressor test time of 2 min).

Throughout the cuff deflation protocol, a transverse scan of the deep and superficial veins was obtained 5–6 cm proximal to the right cubitus in the resting arm using a high‐resolution ultrasound device in B‐mode (Vivid e, GE Healthcare Japan, Tokyo, Japan) with a linear‐array transducer that had a mean transmission frequency of 8.7 MHz. The CSA was calculated by manually tracing the edge of the transverse venous image at intervals of 4 sec (4 mmHg). The relationship between cuff pressure and CSA (i.e., pressure‐CSA curve) was generated from the data points between 60 mmHg and 10 mmHg during the cuff deflation protocol. To avoid any priori assumption on pressure (P)‐CSA curve and obtain the physiological compliance curve, compliance (CPL) was calculated as the numerical derivative of each pressure‐CSA data point pair with the following equation (Freeman et al. 2002).

$${\text{CPL}}_{pi}=\frac{{\text{CSA}}_{i}-{\text{CSA}}_{i-1}}{P_{i}-P_{i-1}}\;\text{where}1\leq i\leq 60$$

The compliance of the brachial vein (CPL_*deep*_) and that of the basilic vein (CPL_*sup*_) were obtained. The coefficient of variation (CV) for pre‐exercise CSA measurements in this study was 4.8 ± 0.6% for CSA_*deep*_ and 2.5 ± 0.3% for CSA_*sup*_.

### Measurements of body temperature, arterial blood flow, and cardiorespiratory variables

Rectal temperature was measured every 2 sec with a thermistor probe that was inserted 10 cm beyond the anal sphincter. Local skin temperature was measured at intervals of 1 sec at six sites using copper‐constantan thermocouples taped to the skin. The mean skin temperature was calculated by weighting the six sites as follows: forehead, 0.14; chest, 0.19; back, 0.19; forearm, 0.11; hand, 0.05; and thigh, 0.32.

Mean arterial pressure (MAP) was measured noninvasively by photoelectric plethysmography using a Finometer (Finapres Medical Systems BV, Amsterdam, The Netherlands). Heart rate (HR) and stroke volume (SV), and hence cardiac output (CO), were determined from the blood pressure waveform using the Modelflow software program, which incorporates sex, age, height, and weight (BeatScope 1.1, Finapres Medical Systems BV).

Blood flow in the right brachial artery (ABF) was measured immediately after assessment of CSA in the vein. Mean blood flow velocity (cm/s) and vessel diameter were measured by pulsed wave mode and B‐mode ultrasound, respectively, with a linear‐array 5–12 MHz probe (Vivid e). The vessel diameter during systole and diastole, judged from the electrocardiographic waveform, was measured to allow calculation of the mean diameter as follows: mean diameter (cm) = (systolic diameter × 1/3) + (diastolic diameter × 2/3). ABF was then calculated as follows: ABF (ml/min) = mean flow velocity × *π *× (mean diameter/2)^2^ × 60 sec. Vascular conductance in the brachial artery (AVC) was calculated as the ratio of ABF to MAP.

For determination of $\dot{V}O_{2\mathrm{peak}}$, the expired gas fractions and airflow were continuously measured using a mass spectrometer (Arco 1000, Arco System, Chiba, Japan). HR was simultaneously monitored during exercise by electrocardiographic recordings from a pair of chest electrodes (Radercirc; Sumitomo Dainippon Pharma Co., Ltd, Osaka, Japan).

### Data analysis and statistics

SV and CO values were normalized in each subject relative to the pre‐exercise level (100%). Rectal temperature, skin temperature, MAP, HR, SV, CO, ABF, and AVC within subjects during the assessment of the deep and superficial vein and the mean value of each variable was used.

Rectal temperature, skin temperature, MAP, HR, SV, CO, ABF, and AVC were compared between pre‐exercise and during exercise using the paired *t*‐test. To compare the changes with cuff pressure between pre‐exercise and during prolonged exercise, two‐way analysis of variance (ANOVA) with repeated measures was applied to the CSA and CPL obtained by a cuff pressure of 10–60 mmHg under each condition (pre‐exercise and during exercise), using cuff pressure and condition as fixed factors. If a main effect of condition and/or interaction was detected, post hoc analysis with a paired t‐test was performed every 4 mmHg. The effect of heat stress and cold pressor test on CSA and CPL was also analyzed as same as prolonged exercise. The statistical analysis was performed using SPSS version 19 software (IBM Corp., Armonk, NY). A *P‐*value < 0.05 was considered to be statistically significant. The data are shown as the mean ± standard error of the mean.

## Results

### Effect of prolonged exercise on CSA and CPL of deep and superficial veins

Table 1 shows body temperature and the cardiovascular variables at rest and during prolonged exercise. All variables were increased by prolonged exercise from pre‐exercise levels.

**Table 1:** Body temperature and cardiovascular variables at pre‐exercise and during exercise

	Pre‐exercise	During exercise
Rectal temperature (°C)	36.8 ± 0.1	37.9 ± 0.1^a^
Mean skin temperature (°C)	33.0 ± 0.2	34.1 ± 0.2^a^
Blood flow of brachial artery (mL/min)	72.4 ± 11.5	302.6 ± 45.4^a^
Vascular conductance of brachial artery (mL/min/mmHg)	0.8 ± 0.1	2.8 ± 0.4^a^
Mean arterial pressure (mmHg)	93 ± 2	107 ± 3^a^
Heart rate (bpm)	66 ± 3	149 ± 4^a^
Stroke volume (%)	100	121.7 ± 2.8^a^
Cardiac output (%)	100	257.2 ± 15.2^a^

Values are means ± SE.

a Significant difference between pre‐exercise and during exercise, *P *<* *0.05.

**Figure 1:**
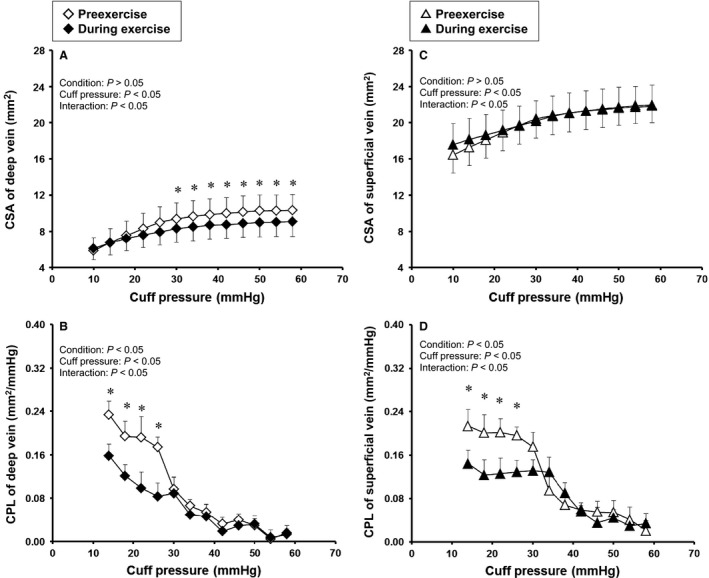
Cuff pressure–cross sectional area (CSA) curves and cuff pressure–compliance (CPL) relations in deep and superficial veins at pre‐exercise and during prolonged exercise. Values are mean ± SE. Probability given by two‐way ANOVA are shown in figures of A–D; Condition, main effect of pre‐exercise and exercise; Cuff pressure, main effect of cuff pressure; Interaction, interaction effects. * Significant difference between pre‐exercise and during exercise in post hoc test, *P *<* *0.05.

Figure 1 shows the effects of prolonged exercise on CSA and CPL in the deep and superficial veins. In the deep vein, the CSA_*deep*_ during exercise was lower than that at pre‐exercise in the higher range of cuff pressure (Fig. 1A). ANOVA yielded a significant interaction effect (*P *<* *0.01), and a post hoc test showed a significant difference in mean CSA_*deep*_ between pre‐exercise and exercise at a cuff pressure in the range of 30–60 mmHg (*P *<* *0.05). The CPL_*deep*_ during exercise (Fig. 1B) was lower than that at pre‐exercise in the lower range of cuff pressure. ANOVA showed significant main and interaction effects (*P *<* *0.05), and a post hoc test showed a significant difference in the mean values obtained between pre‐exercise and exercise at a cuff pressure below 26 mmHg.

In contrast, the CSA_*sup*_ (Fig. 1C) was higher in the superficial vein during exercise at a cuff pressure below 20 mmHg. ANOVA yielded a significant interaction effect, but a post hoc test failed to show a significant difference in the mean CSA_*sup*_ between pre‐exercise and exercise. CPL_*sup*_ (Fig. 1D) decreased significantly during exercise, as was observed for CPL_*deep*_ (Fig. 1B).

### Effect of passive heating on CSA and CPL of deep and superficial veins

T_re_, T_sk_, AVC, and HR were significantly higher in resting period in HOT than in CON (T_re_: 36.8 ± 0.1°C in CON and 37.2 ± 0.1°C in HOT, *P *<* *0.05; T_sk_: 33.0 ± 0.2°C in CON and 35.6 ± 0.2°C in HOT, *P *<* *0.05; AVC: 0.8 ± 0.1 ml/min/mmHg in CON and 1.7 ± 0.2 mL/min/mmHg in HOT, *P *<* *0.05; HR: 66 ± 3 bpm in CON and 75 ± 4 bpm in HOT, *P *<* *0.05). MAP was not different between HOT and CON (93 ± 2 mmHg in CON and 91 ± 2 mmHg in HOT).

**Figure 2:**
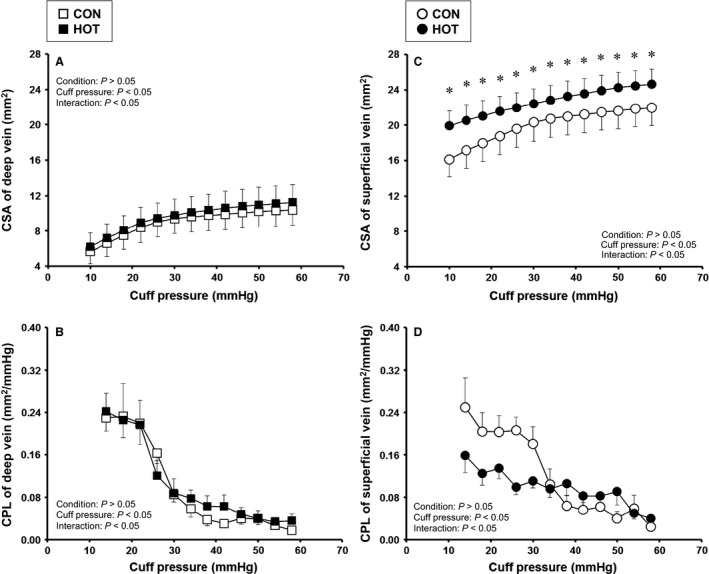
Effect of passive heating without exercise on cross‐sectional area (CSA) and compliance (CPL) in deep and superficial veins. Values are mean ± SE. Probability given by two‐way ANOVA are shown in figures of A–D; Condition, main effect of CON and HOT; Cuff pressure, main effect of cuff pressure; Interaction, interaction effects. * Significant difference between CON and HOT in post hoc test, *P *<* *0.05.

Figure 2 shows the effects of passive heating on CSA and CPL in the deep and superficial veins. In the deep vein, CSA_*deep*_ and CPL_*deep*_ were similar between HOT and CON (Fig. 2A and B). On the other hand, the CSA_*sup*_ was greater in HOT than CON in all range of cuff pressure (*P *<* *0.05, Fig. 2C). The CPL_*sup*_ was smaller in HOT than CON in the lower range of cuff pressure (Fig. 2D). ANOVA yielded a significant interaction effect, but a post hoc test failed to show a significant difference in the mean CPL_*sup*_ between conditions.

### Effect of cold pressor test on CSA and CPL of deep and superficial veins

HR and MAP were significantly higher in cold pressor test than resting condition (HR: 59 ± 3 bpm in rest and 69 ± 4 bpm in cold pressor test, *P *<* *0.05; MAP: 75 ± 2 mmHg in rest and 89 ± 3 mmHg in cold pressor test, *P *<* *0.05).

**Figure 3:**
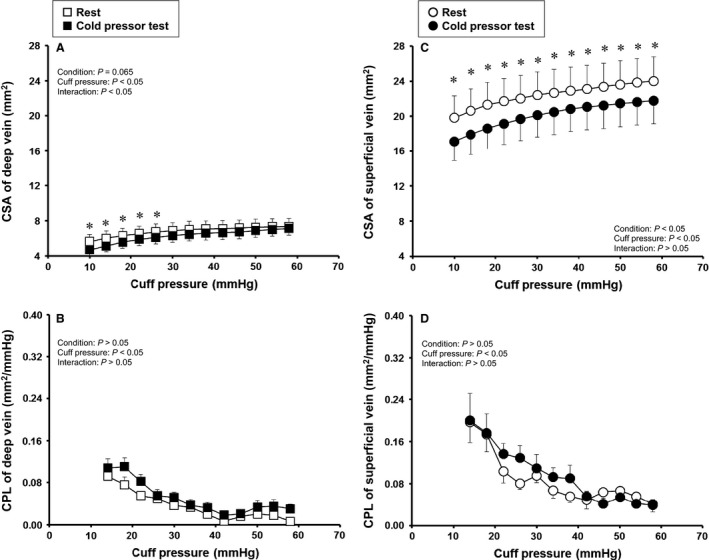
Effect of cold pressor test on cross‐sectional area (CSA) and compliance (CPL) in deep and superficial veins. Values are mean ± SE. Probability given by two‐way ANOVA are shown in figures of A–D; Condition, main effect of rest and cold pressor test; Cuff pressure, main effect of cuff pressure; Interaction, interaction effects. * Significant difference between rest and cold pressor test in post hoc test, *P *<* *0.05.

Figure 3 shows the effects of cold pressor test on CSA and CPL in the deep and superficial veins. In both deep and superficial veins, CSA was smaller in cold pressor test than rest (*P* < 0.05, Fig. 3A and C). On the other hand, CPL in both veins was not different between rest and cold pressor test (Fig. 3B and D).

## Discussion

This study is the first to investigate the responses of CSA and CPL at the level of a single deep and a single superficial conduit vein during prolonged exercise. In addition, to confirm the response of CSA and CPL during prolonged exercise, we investigated the effect of passive heating and cold pressor test on the CSA and CPL of deep and superficial veins. Our new findings are that (1) in spite of opposite responses of CSA between the deep and the superficial veins, the CPL in both conduit veins were similarly reduced during prolonged exercise, (2) passive heating without exercise caused the increased CSA and the decreased CPL of superficial vein when compared with prolonged exercise, but did not change CSA and CPL of deep vein, and (3) cold pressor test, which induced sympathoexcitation, caused the decreased CSA of deep and superficial veins without the alteration of CPL of both veins. These findings suggest different mechanisms for the adjustment of CPL to prolonged exercise between conduit deep and superficial veins.

### Difference in response of CSA between deep and superficial veins during prolonged exercise

Our results showed contrasting responses for CSA in that the CSA_*deep*_ decreased while the CSA_*sup*_ increased during prolonged exercise (Fig. 1). Although there are few data that are directly comparable with our present data, a reduction in CSA_*deep*_ was also observed in our previous study (Ooue et al. 2008) where the diameter of a deep (brachial) vein decreased gradually with an increase in core temperature during prolonged cycling exercise. The reduction in CSA_*deep*_ could have been a byproduct of passive collapse and/or depression in response to the decrease in pressure (Öberg 1967; Noble et al. 1998) caused by decreased blood flow to the muscles in the non‐exercising limb during prolonged exercise (Johnson and Rowell 1975). However, this explanation is unlikely because the ultrasound image of the CSA_*deep*_ showed a circular CSA without depression in the present study. Alternatively, we considered that active venoconstriction induced by sympathetic excitation during exercise was a possible mechanism for the reduction in CSA_*deep*_. At first, previous study reported that the cycling exercise at 60% $\dot{V}O_{2\mathrm{peak}}$ produced a significant increase in sympathetic nerve activity in muscle (Saito et al. 1997), and further, the sympathetic nerve activity occurring during exercise was capable of altering venous CSA via the muscle metaboreflex (Duprez et al. 1992; Ooue et al. 2013) and by central command (Ooue et al. 2012). In addition, the indirect action of circulating norepinephrine could also induce active venoconstriction (Abdel‐Sayed et al. 1970), and dynamic exercise caused the increase in plasma norepinephrine concentration (Christensen and Brandsborg 1973; Lehmann et al. 1985; Mazzeo and Marshall 1989). Although there is less sympathetic innervation in the deep veins than in the superficial veins, deep vein vessels also constricted slightly but significantly by these factors (Abdel‐Sayed et al. 1970). Taken together, the active venoconstriction seen in the present study was probably attributable to the reduction in CSA_*deep*_ during prolonged exercise, even though we cannot separate completely the decreased CSA_*deep*_ into the active and passive venoconstriction because of no mechanistic investigation in present study.

In contrast, an opposite response was found for the CSA_*sup*_ during prolonged exercise. We consider that the increased CSA_*sup*_ was caused by some degree of passive dilation (or distension) due to increased blood flow in the superficial vein, which in turn could have overridden the active venoconstriction that occurred in the superficial vein during prolonged exercise. Support for this explanation can be found in our previous study, which showed that CSA_*sup*_ decreased transiently at the start of exercise and then increased gradually in proportion to the blood flow to the skin in the upper arm during 30 min of cycling exercise (Ooue et al. 2008). As well as passive dilation, the active venodilation might also effect on the increase in CSA_*sup*_, since superficial vein vessel is also capable of dilating actively due to endothelium‐derived relaxing factors (cf. NO etc.) (Collier and Vallance 1989; Vallance et al. 1989; Rabelo et al. 2008). However, we consider that the effect of active dilation is a little. If the active venodilation, which was caused by the relaxation of smooth muscle, contributed mainly to the increased CSA_*sup*_, CPL_*sup*_ was expressed to increase. Indeed, however, it does not so in this study. Thus, although the superficial veins are richly innervated by sympathetic nerves (Bevegard and Shepherd 1965; Rowell et al. 1971; Zitnik et al. 1971), passive dilation in response to markedly increased venous blood flow during prolonged exercise probably caused the increased CSA_*sup*_, with masking of active venoconstriction.

### Similar decrease in compliance in deep and superficial veins during prolonged exercise

Both CPL_*deep*_ and CPL_*sup*_ decreased during prolonged exercise despite the opposite responses seen in CSA_*deep*_ and CSA_*sup*_ (Fig. 1). The effect of sympathetic activation with venoconstricition on venous compliance is not conclusive. Some studies have reported that the sympathoexcitation during post‐exercise muscle ischemia, adrenergic blockade or norepinephrine administration caused the decrease in venous unstressed volume without changed compliance (Greenway et al. 1985; Halliwill et al. 1999; Sielatycki et al. 2011). In addition, the change in CSA and CPL during cold pressor test (Fig. 2) also supported these previous studies. On the other hand, other studies have indicated that the sympathoexcitation during hemorrhage or lower body negative pressure induced the decrease in not only unstressed volume but also compliance in vein (Rothe and Drees 1976; Shoukas and Bohlen 1990; Monahan and Ray 2004). The different venous compliance response between studies might be due to the contribution of the cardiopulmonary and arterial baroreflexes, because it is speculated that the remarkable decrease in central blood volume occurred in the latter but not former studies. In addition, previous study showed that prolonged exercise caused the increase in muscle sympathetic nerve activity via the unloading of cardiopulmonary receptor(s) according to the increase in sweating and skin blood flow (Saito et al. 1997). Considering these, although we cannot exclude completely the passive decrease in venous volume, it is speculated that the decrease in CPL of deep vein obtained during prolonged exercise in our study might result from, in part, the active venoconstriction which was specific to the sympathoexcitation via baroreceptor(s) unloading. In contrast, for the decrease in CPL_*sup*_, we suggest that passive dilation, which probably explained the increase in CSA_*sup*_, simultaneously stretched (or strained) the vessel wall, thereby decreasing the CPL_*sup*_ during prolonged exercise. These explanations are in line with previous reports found that when vessel is actively constricted (Shoukas and Bohlen 1990) or passively stretched (Holman et al. 1968; Johansson and Mellander 1975), contractile properties of smooth muscle in the vessel become stiff. Thus, the similar decrease in CPL_*deep*_ and CPL_*sup*_ in the present study suggests different mechanisms for the adjustment of CPL to prolonged exercise in that the decrease in CPL_*deep*_ was probably mediated by active venoconstriction while the decrease in CPL_*sup*_ was attributable to passive dilation of the vessel.

This study has some limitations. First, although the CV for CSA measurements in this study was lower than that in previous studies that have measured CSA in the basilic vein (Ooue et al. 2013) and popliteal vein (de Groot et al. 2005), there are measurement errors in the assessment of CSA of a single vein by ultrasonography. Second, measurement of CSA in this study was based on a report (Halliwill et al. 1999) showing that the collecting cuff pressure was nearly equal to the intravenous pressure, but we did not verify intravenous pressure directly in our study. Third, we acknowledge the limitation of discussing active venoconstriction and passive venodilation in the absence of precise mechanisms. Finally, we did not investigate the change in venous compliance during prolonged exercise in female subjects in present study, even though the venous compliance has been shown to differ between male and female (Lindenberger and Länne 2007), which seems due to the different sex hormones and body fluid homeostasis (Stachenfeld and Taylor 2005). However, in our previous studies (Ooue et al. 2007, 2008, 2013), the pattern of change in CSA and blood flow of veins during physiological stress was almost similar between female and male. Thus, it is speculated that the decreased venous compliance during prolonged exercise occur in not only male but also female. We have to investigate this point furthermore. Even though these limitations remained to be resolved, we believe that our present findings regarding compliance at the level of individual conduit veins provide a new insight into adjustment of the venous system during exercise.

## Conclusions

This study demonstrates that CPL in the deep and superficial conduit veins were similarly reduced during prolonged exercise in spite of opposite CSA responses. We suggest that the reduction in CPL in the deep vein was mediated by active venoconstriction while that in the superficial vein was by passive dilation of the vessel due to increased blood flow.

## Conflict of Interest

The authors have no financial conflict of interest .
